# Sex Education in the Spotlight: What Is Working? Systematic Review

**DOI:** 10.3390/ijerph18052555

**Published:** 2021-03-04

**Authors:** María Lameiras-Fernández, Rosana Martínez-Román, María Victoria Carrera-Fernández, Yolanda Rodríguez-Castro

**Affiliations:** Faculty of Education and Social Work, University of Vigo, 32004 Ourense, Spain; rosana.mr@uvigo.es (R.M.-R.); mavicarrera@uvigo.es (M.V.C.-F.); yrcastro@uvigo.es (Y.R.-C.)

**Keywords:** adolescents, sexual education, sexual and reproductive health, review of reviews, school setting, digital platforms, blended learning

## Abstract

Adolescence, a period of physical, social, cognitive and emotional development, represents a target population for sexual health promotion and education when it comes to achieving the 2030 Agenda goals for sustainable and equitable societies. The aim of this study is to provide an overview of what is known about the dissemination and effectiveness of sex education programs and thereby to inform better public policy making in this area. *Methodology*: We carried out a systematic review based on international scientific literature, in which only peer-reviewed papers were included. To identify reviews, we carried out an electronic search of the Cochrane Database Reviews, ERIC, Web of Science, PubMed, Medline, Scopus and PsycINFO. This paper provides a narrative review of reviews of the literature from 2015 to 2020. *Results*: 20 reviews met the inclusion criteria (10 in school settings, 9 using digital platforms and 1 blended learning program): they focused mainly on reducing risk behaviors (e.g., VIH/STIs and unwanted pregnancies), whilst obviating themes such as desire and pleasure, which were not included in outcome evaluations. The reviews with the lowest risk of bias are those carried out in school settings and are the ones that most question the effectiveness of sex education programs. Whilst the reviews of digital platforms and blended learning show greater effectiveness in terms of promoting sexual and reproductive health in adolescents (ASRH), they nevertheless also include greater risks of bias. *Conclusion*: A more rigorous assessment of the effectiveness of sexual education programs is necessary, especially regarding the opportunities offered by new technologies, which may lead to more cost-effective interventions than with in-person programs. Moreover, blended learning programs offer a promising way forward, as they combine the best of face-to-face and digital interventions, and may provide an excellent tool in the new context of the COVID-19 pandemic.

## 1. Introduction

Adolescence is a period of transition, growth, exploration and opportunities that the World Health Organization defines as referring to individuals between 10 years and 19 years of age [1]. During this life phase, adolescents undergo physical, psychological and sexual maturation and tend to develop an increased interest in sex and relationships, with positive relationships becoming strongly linked to sexual and reproductive health as well as overall wellbeing [2]. Sexual health is understood as a state of wellness comprising physical, emotional, mental, and social dimensions [3]: it represents one of the necessary requirements to achieve the general objective of sustainable and equitable societies in terms of the 2030 Agenda [4], which advocates the need for a sexual education that is anchored in a gender- and human rights-oriented perspective.

In high-income countries, sexual debut usually occurs during adolescence [5], though research suggests that sexual initiation is increasingly occurring at earlier ages [6]. Adolescents have to deal with the results of unhealthy sexual behaviors, including unplanned pregnancies and sexually transmitted infections [7], as well as experiences of sexual violence [8,9]. Adolescents are aware that they need more knowledge in order to enjoy healthy relationships [10], yet do not receive enough of the kind of information from parents or other formal sources that would allow them to develop a more positive, respectful experience of sexuality and sexual relationships [11].

Sexual education can be defined as any combination of learning experiences aimed at facilitating voluntary behavior conducive to sexual health. Sex education during adolescence has centered on the delivery of content (abstinence-only vs. comprehensive instruction) by teachers, parents, health professionals or community educators, and on the context (within school and beyond) of such delivery [12]. As regards content, the proponents of abstinence-only programs aim to help young adults avoid unintended pregnancies and sexually transmitted diseases (STDs), working on the assumption that while contraceptive use merely reduces the risk, abstinence will eliminate it entirely [13]. Nevertheless, an overwhelming majority of studies in this field have shown that programs advocating abstinence-only-until-marriage (AOUM) are neither effective in delaying sexual debut nor in changing other sexual risk behaviors [14,15], and participants in abstinence-only sex education programs consider that these had only a low impact in their lives [16]. On the other hand, holistic and comprehensive approaches to sex education go beyond risk behaviors and acknowledge other important aspects, as for example love, relationships, pleasure, sexuality, desire, gender diversity and rights, in accordance with internationally established guidelines [17], and with the 2030 Agenda [4]. Comprehensive Sexuality Education (CSE) “plays a central role in the preparation of young people for a safe, productive, fulfilling life” (p. 12) [17] and adolescents who receive comprehensive sex education are more likely to delay their sexual debut, as well as to use contraception during sexual initiation [18]. Comprehensive sexual education initiatives thereby promote sexual health in a way that involves not only the biological aspects of sexuality but also its psychological and emotional aspects, allowing young people to have enjoyable and safe sexual experiences.

With regard to context, sexual education may occur in different settings. School settings are key sites for implementing sexual education and for promoting adolescent sexual health [19], but today internet is becoming an increasingly important source of information and advice on these topics [20]. Access to the internet by adolescents is almost universal in high-income countries. The ubiquity and accessibility of digital platforms result in adolescents spending a great deal of time on the internet, and the search for information is the primary purpose of health-related internet use [21]. At the same time, this widespread use of technology by young people offers interesting possibilities for sexual health education programs, given the ease of access, availability, low cost, and the possibility of participating remotely [22]. The topics that young people search for online include information on everyday health-related issues, physical well-being and sexual health [23]. The majority of internet users of all ages in the US (80%) search online for health information including sexual health information [24], and among adolescents social media platforms are the most frequent means of obtaining information about health, especially regarding sexuality [25]. 

Thanks to the ubiquity and popularity of technologies, digital media interventions for sexual education offer a promising way forward, both via the internet (eHealth) and via mobile phones (mHealth, a specific way of promoting eHealth), given the privacy and anonymity they afford, especially for young people. Digital interventions in school—both inside and outside the classroom—offer interesting possibilities, because of their greater flexibility with regard to a variety of learning needs and benefits in comparison with traditional, face-to-face interventions, and because they offer ample opportunities for customization, interactivity as well as a safe, controlled, and familiar environment for transmitting sexual health knowledge and skills [26]. As Garzón-Orjuela et al. [27] argues, contemporary adolescents’ needs are mediated by their digital and technological environment, making it important to adapt interventions in the light of these realities. Online searches for sexual health information are likely to become increasingly important for young people with diminishing access to information from schools or health care providers in the midst of the lockdowns and widespread school closures during the COVID-19 pandemic [28], with more than two million deaths and 94 million people infected around the world [29]. Specifically, blended learning programs, consisting of internet-based educational interventions complemented by face-to-face interventions, may prove a significant addition to regular secondary school sex education programs [30,31]. Blended learning programs can be especially helpful in promoting sexual and reproductive health in the context of the COVID-19 pandemic, which is challenging the way we have so far approached formal education, with its focus on face to face interventions, given the need, now more than ever, to “develop and disseminate online sex education curricula, and ensure the availability of both in-person and online instruction in response to school closures caused by the pandemic” [28].

The present study sets out to research the dissemination and effectiveness in different settings (school, digital and blended learning) of sex education programs that promote healthy and positive relationships and the reduction of risk behaviors, so as to make current sexual health interventions more effective [32]. Numerous researchers have carried out trials and systematic reviews so as to evaluate the effectiveness of school-based sexual health and relationship education [19,27,33,34,35], as well as that of digital platform programs [36,37,38,39]. However, there has not been a review that is representative of the literature as a whole. Furthermore, in the reviews that have been carried out, differing aims and inclusion criteria have led to differences in the sampling of available primary studies [19]. As Garzón-Orjuela et al. [27] asserts, the field of adolescent sex education is continuously evolving and in need of evaluation and improvement. Better assessments are necessary in order to clarify whether they offer a viable and effective strategy for influencing adolescents, especially with respect to improved ASRH behaviors. Hence, given the need for an up-to-date revision so as to consider more recent emerging evidence in this field, in this study we carry out a review of reviews that includes reviews of interventions both in school settings and via digital platforms, as well as, for the first time, those that combine both formats (blended learning). 

The decision to conduct a review of reviews (RoR), assessing the quality and summarizing the findings of existing systematic reviews, rather than working directly with primary intervention studies, addresses the need to include as wide a range of topics covered within the field of sex education as possible [40]. As Schackleton et al. [35] (p. 383) point out, in order to provide overviews of research evidence that are relevant to policy making, it is important “to bring together evidence on different forms of intervention and on different outcomes because it is useful for policy makers to know what is the range of approaches previously evaluated and whether these have consistent effects across different outcomes.” Carrying out and publicly sharing reviews of reviews such as the present study constitutes one way of better providing practitioners with evidence they can then carry over into their interventions [32].

## 2. Methodology

### 2.1. Aims

(1) To systematically review existing reviews of Sex Education (SE) of school-based (face-to-face), digital platforms and blended learning programs for adolescent populations in high-income countries.

(2) To summarize evidence relating to effectiveness.

### 2.2. Methods

**Figure A1:**
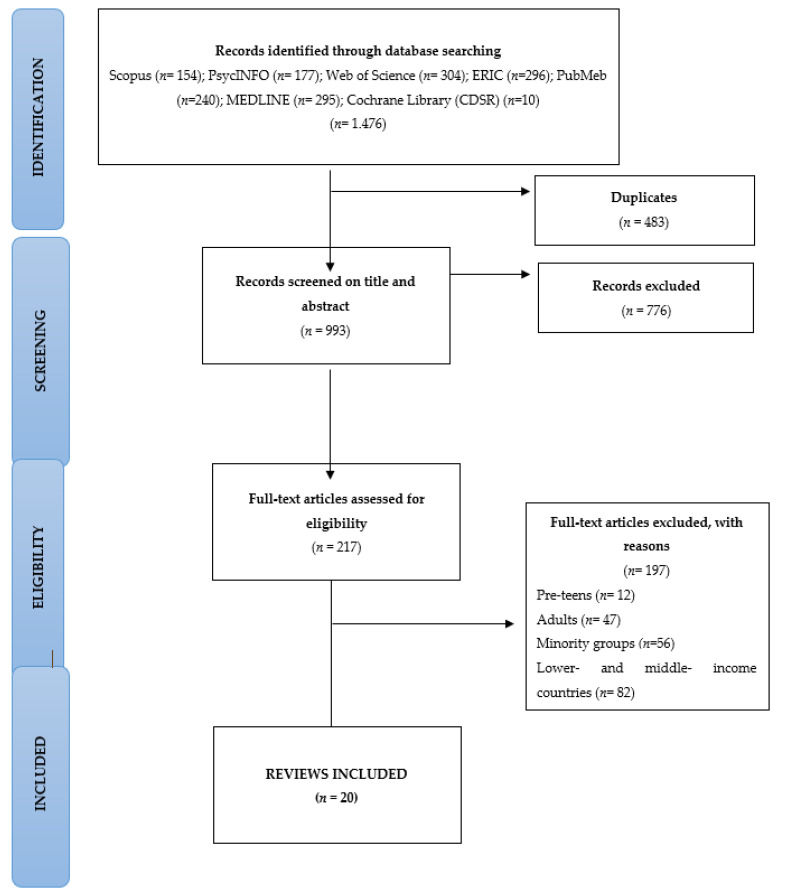
Flow diagram Preferred reporting items for systematic reviews and meta-analysis, PRISMA).

The review is structured in accordance with the PRISMA checklist (Preferred Reporting Items for Systematic Reviews and Meta-Analysis) (see Figure A1), and the systematic review protocol has previously been published on the PROSPERO International Prospective Registry of Technical Reviews (CRD42021224537).

### 2.3. Search Strategy

This systematic review is based on international scientific literature and only peer- reviewed papers have been included. Only meta-analyses (publications that combine results from different studies) and systematic reviews (literature reviews that synthesize high-quality research evidence) were used for this review. Findings from reviews of reviews were not analyzed. To identify reviews, we electronically searched the Cochrane Database Reviews, ERIC, Web of Science, PubMed, Medline, Scopus and PsycINFO. After the list was completed the duplicated papers were automatically removed. Two reviewers working independently applied inclusion criteria in screening citations by titles, abstracts, and keywords to identify records for full-text review. A third reviewer reconciled any disagreement. The same procedure was carried out in screening the full text of studies selected after the title and abstract screening phase. Two reviewers then examined the full text of each article to determine which satisfied inclusion criteria. Data extraction was carried out independently by the first and second reviewer. The extracted data included specific details about the interventions, populations, study methods and outcomes significant to the review question and objective. Any discrepancies were discussed until consensus was reached. Search terms are included in Table A1.

This RoR included the reviews published since 2015, when the United Nations decided on new Global Sustainable Development Goals, until December 2020. The 2030 Agenda for Sustainable Development [4] takes into account the relevance of Sexual Health to achieve peace and prosperity.

### 2.4. Inclusion Criteria

We extracted data using a “Population, Intervention, Comparison, Outcome” structure, PICO [41].

Population: Reviews of interventions targeting adolescents (aged 10–19 years), school-setting, digital platforms or blended learning education were eligible for inclusion. Reviews in which studies of interventions targeted youth and adults were eligible if the primary studies included people between the ages of 10–19 years.

Intervention: Reviews of interventions developed in school-setting (school-based), digital (digital platforms) or blended learning programs were included. Interventions based on multiple settings or targeted multiple health-related issues were only considered for inclusion if any primary studies were linked to school-based, digital or blended learning interventions, as well as targeting Sexual and Reproductive Health (SRH).

Comparison groups: Randomized controlled trials (RCTs) and studies using a quasi-experimental design (including non-randomized trials—nRCTs). Single group, pre- and post-test research designs, group exposed to sexual education (SE) program (school-based, digital platforms or blended learning) compared with non-exposed control group or another intervention.

Outcomes: Primary outcomes: (1) Sexual behavior and (2) Health and social outcomes related to sexual health. Secondary outcomes: (1) Knowledge and understanding of sexual health and relationship issues and (2) Attitudes, values and skills.

### 2.5. Exclusion Criteria

Reviews were excluded if:

- Their primary focus was adult people and adolescents were not included.
- Their primary focus was sexual-health screening, sexual abuse or assault or prevention of sexual abuse or rape.
- The studies targeted specific populations (e.g., pre-pubertal children, children with developmental disorders, migrant and refugee, or sexual minorities).
- The interventions focused on low- and middle-income countries or if high income countries were not included in the study.
- Recipients were professionals, teachers, parents or a combination of the latter.

### 2.6. Risk of Bias and Assessment of Study Quality

Review quality was assessed by the first author using the AMSTAR II checklist [42]. This is an updating and adaptation of AMSTAR [43,44] which allows a more detailed assessment of systematic reviews that include randomized or non-randomized studies of healthcare interventions, or both. It consists of a 16-item tool (including 5 critical domains) assessing the quality of a review’s design, its search strategy, inclusion and exclusion criteria, quality assessment of included studies, methods used to combine the findings, likelihood of publication bias and statements of conflict of interest. The maximum quality score is 16.

### 2.7. Data Synthesis

After manually coding the papers and extracting relevant data, we used a narrative/descriptive approach for data synthesis to summarize characteristics of the studies included. Considering the heterogeneity of outcomes, their measures and research designs, meta-analysis of all the studies included was not carried out. Two researchers were involved in data synthesis. Discrepancies were resolved through discussion, and a third researcher was consulted to resolve any remaining discrepancies. For the classification of the information and presentation of the effects of the interventions reported, data was separated (school setting, digital platforms or blended learning) and structured around population, intervention, comparison, and outcome. To address the main review questions, data was synthesized in two phases. Phase 1 addressed the first question, the description of sex education/sexual health interventions. Phase 2 addressed the second question, the effectiveness and benefit of the interventions; studies with a low risk of bias were highlighted, so as to strengthen the reliability of findings (AMSTAR II) [42].

## 3. Results

### 3.1. Results of Search

Our searches yielded 1476 unique citations. After excluding 776 records based on title and abstract screening, we reviewed 217 full-text articles for eligibility, of which 20 ultimately met inclusion criteria, and proceeded to data extraction. Of the 197 studies that we excluded after full-text review, 82 were carried out in low- and middle-income countries, 47 targeted exclusively adults, 56 dealt with minority groups, and 12 targeted exclusively pre-teen students.

### 3.2. Risk of Bias in Included Studies

According to the AMSTAR II quality assessment tool’s developers [42] scores may range from 1 to 16: in this case only 2 reviews scored 16 out of 16: 1 in a school setting [45], and 1 on a digital platform [46]. 6 of the 20 systematic reviews were of high quality: 5 in school settings [45,47,48,49,50], and 1 in digital platforms [46]; there was one study of medium quality in a school setting [51]. The remaining studies were of low or very low quality (N = 13). It is possible that low quality reviews may not provide reliable evidence, so those scoring in low and critically low quality should be regarded skeptically.

### 3.3. Reviews Included

Key information regarding the 20 reviews included is shown in Table A2 and Table A3.

#### 3.3.1. Setting

Ten studies (50%) dealt with school-based interventions [45,47,48,49,50,51,52,53,54,55], 9 (45%) referred to online interventions [46,56,57,58,59,60,61,62,63] and 1 (5%) was a review of blended learning programs [64]. In total 491 studies were included in the 20 reviews covered by the present RoR. The 10 reviews of school setting interventions include a total of 266 studies (54%), the 9 reviews of online interventions cover a total of 216 (44%) studies, and the only review of blended learning interventions includes a total of 9 studies (2%). All studies were conducted in high-income economies following the World Bank classification [65], including US samples in 16 of the 20 studies, although there are two studies in which the country of the sample is not identified [51,52]. Most of the studies evaluating interventions in school settings also include developing countries (low- and middle-income economies) [45,47,50,52,53,55], as is also the case in three reviews of online interventions [46,61,62] (see Table A2).

#### 3.3.2. Population

The targeted age for reviews in school settings, as shown in Table A2, is the period of adolescence, from 10 to 19 years of age, though one of the studies covers ages from 7 to 19 years [53]. All the online studies also include young adults (20–24 years old), alongside the adolescent sample [46,56,57,59,60,61,62,63], whilst the review by DeSmet et al. [58] extends the upper limit to 29 years of age. Along with the sample of adolescents and young adults, the blended learning studies review also incorporates adults of over 25 years of age [64].

#### 3.3.3. Interventions/Types of Study

All the studies included in this review of reviews used randomized controlled trials (RCTs), non-randomized controlled trials (non-RCT), and a quasi-experimental design or a pre-test/post-test design to examine program effects.

#### 3.3.4. Outcomes

The term “sexual outcomes” refers to the attitudes, behaviors, and experiences of adolescents consequent to their sex education [14] (p. 1), and an extensive range of variables was included (see Table A2): knowledge (e.g., knowledge of contraceptive effectiveness or effective method use); attitudes (e.g., about sex and reproductive health); beliefs (e.g., self-efficacy); skills (e.g., condom skills); intentions/motivation (e.g., use of birth control methods; condom use); behaviors (e.g., sexual debut; condom use; contraception use; intercourse; initiation of sexual activity) and; other outcomes related to sexual behavior (e.g., pregnancy prevalence; number of partners; rates of sexually transmissible infections (STIs); cervical screening; appreciation of sexual diversity; dating and intimate partner violence prevention; sexual violence).

#### 3.3.5. Country of Review

Of the 10 reviews of interventions in school settings, the authors are from the USA in 7 reviews [47,48,49,50,53,54,55], from the United Kingdom in 1 [45], from Australia in 1 [51], and from Thailand in 1 [52]. Of the 9 reviews of interventions in digital settings, the authors are from the United States in 3 reviews [59,60,63], from the United Kingdom in 2 [46,56], from Australia in 1 [62], from Belgium in 1 [58], from France in 1 [61] and from Turkey in 1 [57]. The authors of the blended learning review are from the USA [64].

#### 3.3.6. Year of Last Paper Included

The studies cited in the reviews that met the inclusion criteria for this review were published over a wide range of years (between 1981–2019), although only one [61], with articles published up to and including 2019 was published later than 2017. Of these, 3 were carried out in school settings [49,51,53], and 1 on digital platforms [46].

#### 3.3.7. Search Tools

All reviews include more than 2 tools to carry out the search, in a range of 3–12, and in 7 of them the review of gray literature was included.

#### 3.3.8. Multicenter Studies and Number of Studies Included

All reviews from school settings are multicenter, except that of Mirzazadeh et al. [49], which includes only one North American sample. The same is true for the blended learning review [64] and for the reviews of digital platforms, except for the reviews by Bailey et al. [56], L´Engle et al. [60], and Widman et al. [63]. Regarding the number of countries included in the reviews, the range in the school-setting reviews is from 1 to 11, in digital platforms reviews from 1 to 16, and in the only review of blended learning, 3. As for the range of studies included, in the reviews in school setting the range is between 8 and 80, in digital platforms, between 5 and 60, and in the only review of reviews of blended learning 9 studies were included.

#### 3.3.9. Number of Reviews Covered That Include Meta-Analysis

As for the number of reviews that carry out a meta-analysis, there are 8 in total: 4 in school settings [45,48,49,55] and 4 on digital platforms [43,46,56,58], while in the only review of blended learning there is no meta- analysis.

### 3.4. Effectiveness

#### 3.4.1. School Settings

Half of the reviews conclude that interventions are not effective in promoting healthy sexual behaviors and/or reducing risks [45,47,48,49,50]. These reviews are of high quality and with a reduced risk of bias (see Table A4), so that the results are highly reliable, even though in most of the studies cited the risk of bias was judged to be high and the quality of evidence was low or very low. These reviews include those of the Marseille et al. [48] and Mirzazadeh et al. [49] team, who in two studies—each led by one of the two authors—analyze, on the one hand, the effectiveness of school-based teen pregnancy prevention programs [48], and, on the other hand, the effectiveness of school-based programs prevent HIV and other sexually transmitted infections in North America [49]. The results of the studies question the usefulness of interventions carried out in schools to prevent both unwanted pregnancies and the incidence of HIV and other sexual transmitted infections in adolescents in North America. In addition to these results, those of Lopez et al. [47] focus on analyzing the effectiveness of programs implemented in schools to promote the use of contraceptive methods and conclude that many trials reported contraceptive use as an outcome but did not take into consideration whether contraceptive methods and their relative effectiveness were part of the content. For its part, the review by Mason-Jones et al. [45] also concludes that the educational programs covered had no significant effect as regards the prevalence of HIV or other STIs (herpes simplex virus, moderate evidence and syphilis, low evidence), nor was there any apparent effect in terms of the number of pregnancies at the end of the trial (moderate evidence). Finally, the review by Oringanje et al. [50] finds only limited evidence for program effects on biological measures, and inconsistent results for behavioral (secondary) outcomes across trials and concludes that it was only the interventions which combined education and contraception promotion (multiple interventions) that led to a significant reduction in unintended pregnancies over the medium- and long-term follow-up period.

In contrast to these negative results in terms of the effectiveness of the programs implemented in the school environment (identified in 5 of the 10 reviews included), 3 of the 10 reviews concluded that the programs evaluated were mostly effective in promoting knowledge, attitudes and/or in reducing risk behaviors [51,52,53] whilst programs were effective in terms of some of the primary outcomes in the reviews by Haberland et al., [54], and Peterson et al. [55]. However, these data must be taken with caution since the level of bias in these reviews—excepting that of Kedzior et al. [51] with a medium quality level—is at a low or critically low-quality level. In the review by Chokprajakchad et al. [52], 22 programs reviewed were effective in changing targeted adolescent psychosocial and/or behavioral outcomes, in 12 of 17 studies evaluating delay in the initiation of sexual intercourse, the programs were effective and many of the reviewed studies demonstrated impacts on short-term outcomes, such as knowledge, attitudes, perception and intention. The review by Goldfarb et al. [53] identifies changes in appreciation of sexual diversity, dating and intimate partner violence prevention, healthy relationships, child sex abuse prevention and additional outcomes. According to the review by Kedzior et al. [51], focused on studies promoting social connectedness with regard to sexual and reproductive sexual health, the programs reviewed improved condom use, delayed initiation of sex, and reduced pregnancy rates. Additionally, in this review, program effectiveness was influenced by ethnicity and gender: greater improvements in condom use were often reported among African American students. For its part, in the study by Peterson et al. [55] the meta-analysis of three randomized trials provided some evidence that school-environment interventions may contribute to a later sexual debut while their narrative synthesis of other outcomes offered only mixed results.

Finally, the review by Haberland et al. [54], which focused on studies analyzing whether addressing gender and power in sexuality education curricula is associated with better outcomes, concluded that where interventions addressed gender or power (N = 10/22) there was a fivefold greater likelihood of effectiveness than in those that did not.

#### 3.4.2. Online Platforms

The reviews included show a very diverse panorama of digital platforms used to carry out educational interventions (e.g., websites, social media, gaming, apps or text messaging and mailing), which makes it difficult to compare the results. Of the 9 reviews of studies included, only one—in which the effects of TCCMD (Targeted Client Communication delivered via Mobile Devices) are evaluated [46]—meets the quality criteria according to the AMSTAR II quality assessment tool [42] (see Table A4); the rest include biases that limit the reliability of the results so that these must be taken with caution. In the studies reviewed by Palmer et al. [46] among adolescents nine programs were delivered only via text messages; four programs used text messages in combination with other media (for example, emails, multimedia messaging, or voice calls); and one program used only voice calls.

When compared with more conventional approaches, interventions that use TCCMD may increase sexual health knowledge (low certainty evidence), and may modestly increase contraception use (low certainty evidence) while the effect on condom use remains unclear given the very low certainty evidence. Additionally, when compared with digital non-targeted communication, the effects TCCMD on sexual health knowledge, condom and contraceptive use are also unclear, again given the very low-certainty evidence. The review finds evidence of a modest beneficial intervention effect on contraceptive use among adolescent (and adult) populations, but that there was insufficient evidence to demonstrate that this translated into a reduction in contraception.

Most of the reviews included refer to changes to a greater or lesser extent [56,57,59,60,62,63], while no changes determined by the intervention were identified in the study by DeSmet et al. [58]. Finally, the review by Martin et al. [61] does not include details about changes as a result of the programs.

The review by L´Engle et al. [60] assesses mHealth mobile phone interventions for ASRH (almost all of which were carried out via SMS platforms, with the notable exception of only four of the programs covered which used other media formats instead of or as well as SMS). The interventions reviewed set out to foster positive and preventive SRH behaviors, augment take-up and continued use of contraception, support medication adherence for HIV-positive young people, support teenage parents, and encourage use of health screening and treatment services. Results from the studies covered in the review offer support for diverse uses of mobile phones in order to help further ASRH. The health promotion programs that made use of text messaging demonstrated robust acceptability and relevance for young people globally and contributed to improved SRH awareness, less unprotected sex, and more testing for STIs. However, the review also found that improved reporting on essential mHealth criteria is necessary in order to understand, replicate, and scale up mHealth interventions. Holstrom’s [59] review, focused on evaluations of internet-based sexual health interventions, finds that these were associated with greater sexual health knowledge and awareness, lower rates of unprotected sex and higher rates of condom use, as well as increased STI testing. Moreover, the review explores young people’s continuing use of and trust in internet as a source of information about sexual health, as well as the particular themes that interest them. Specifically, the study finds that young people want to know not only about STIs, but also about sexual pleasure, about how to talk with partners about their sexual desires, as well as about techniques to better pleasure their partners.

The review by Widman et al. [63] reveals a significant weighted mean effect of technology-based interventions on condom use and abstinence, the effects of which were not affected by age, gender, country, intervention, dose, interactivity, or program tailoring. The effects were more significant when evaluated with short-term (one to five months) follow-ups than with longer term (over six months) ones. Moreover, digital programs were more effective than control programs in contributing to sexual health knowledge and safer sex norms and attitudes. This meta-analysis, drawing on fifteen years of research into youth-oriented digital interventions, is clear evidence of their ability to contribute to safer sex behavior and awareness. In the review by Wadham et al. [62] the majority of studies used a web-based platform for their programs (16 out of 25). These web-based programs varied between complex, bespoke multimedia interventions to more simplified educational modules. Five studies employed SMS platforms both via mobile phone messaging and web-based instant message services. Three of the programs used social networking sites, either for live chat purposes or alongside a web-based platform. Several studies showed that variety in terms of media and platforms was associated with stronger positive responses among participants and improved outcomes. Eleven of the twenty-five studies focused specifically on HIV prevention, with seven finding a statistically significant effect of the program with regard to knowledge levels about prevention of HIV and other STIs, as well as about general sexual health knowledge. However, only twenty percent of the programs that assessed intended use of condoms reported significant effects due to the intervention.

The review by Bailey et al. [56] (p. 5) assesses interactive digital interventions (IDIs), defined as “digital media programs that provide health information and tailored decision support, behavioral-change support and/or emotional support” and focuses on the sexual well-being of young people between the ages of thirteen and twenty four in the United Kingdom. IDIs have significant though small effects on self-efficacy and sexual behavior, although there is not sufficient evidence to ascertain the effects on biological outcomes or other longer-term impacts. When comparing IDIs with in-person sexual health programs, the former demonstrate significant, moderate positive effects on sexual health knowledge, significant small effects on intention but no demonstrable effects on self-efficacy. The review by Celik et al. [57] looks at digital programs (the majority internet- and computer-based with only six making use of mobile phone-based applications) and sets out to understand their effectiveness in changing adolescents’ health behaviors. Findings from the studies (*n* = 9) suggest that the digital interventions carried out with the adolescents generally had a positive effect on health-promoting behaviors. However, in another study focused on fostering HIV prevention [66], there was a statistically significant increase in health-promoting behavior in only one of the four studies reviewed.

In the review by DeSmet et al. [58], no significant behavioral changes as a result of the interventions for sexual health promotion using serious digital games are identified, although the interventions did have significant though small positive effects on outcomes. The fact that so few studies both met the inclusion criteria and also analyzed behavioral effects suggests the need to further investigate the effectiveness of this kind of game-based approach.

Finally, in the review by Martin et al. [61] 60 studies were covered, detailing a total of 37 interventions, though only 23 of the reviews included effectiveness results. A majority of the interventions were delivered via websites (*n* = 20) while online social networks were the second most favored medium (*n* = 13), mostly via Facebook (*n* = 8). The programs under review favored online interaction, principally amongst peers (*n* = 23) but also with professionals (*n* = 16). The review concludes that ASHR programs promoting these kinds of online participation interventions have demonstrated feasibility, practical interest, and attractiveness, though their effectiveness has yet to be determined, given that they are still in the early stages of design and evaluation.

#### 3.4.3. Blended Learning

In the only blended learning review included in our study [64], the authors conclude that blended learning approaches are being successfully applied in ASHR interventions, including in school-based programs, and have led to positive behavioral and psychosocial changes. However, these results should be treated with caution as the review does not follow the guidelines recommended in the AMSTAR II quality assessment tool [44] (see Table A4) and only includes nine studies.

## 4. Discussion

The present review of reviews assesses, for the first time jointly to our knowledge, the effectiveness of sexual education programs for the adolescent population (ASRH) developed in school settings, digital platforms and blended learning. Of the twenty reviews included (comprising a total of 491 programs, mostly from the USA), ten correspond to reviews of programs implemented in school settings, nine to those dealing with interventions via digital platforms and only one deals with studies relating to blended learning. Twelve (60%) of the reviews included (6 out of 10 in school settings, 5 out of 9 on digital platforms, and the only blended learning review) have been published in the last 3 years (between 2018 and 2020). Thus, the present study constitutes the most up-to-date and recent review of reviews incorporating several contemporary studies not covered by earlier reviews [19,27,33,35,36,37,38,39].

### 4.1. Interventions Reviewed

The interventions included in the reviews covered by our study were largely focused on reducing risk behaviors (e.g., VIH/STIs and unwanted pregnancies), and envisaging sex as a problem behavior. Programs reviewed often focused on the physical and biological aspects of sex, including pregnancy, STIs, frequency of sexual intercourse, use of condom, and reducing adolescents´ number of sexual partners. One exception is Golfard’s et al. [53] review about comprehensive sex education, which is centered on healthy relationships and sexual diversity, though it also makes reference to prevention of violence (dating and intimate partner violence prevention and sex abuse prevention). However, Golfard’s et al.’s [53] rejects more than 80% of the studies initially reviewed because they were focused solely on pregnancy and disease prevention. In the reviews of interventions on digital platforms and via blended learning all the outcomes focused on behaviors related to sexual health (focused on the prevention of risk behaviors), and in several cases also addressed perceived satisfaction and usability. These results are in line with other studies that confirm the over-attention given to risk behaviors, to the detriment of other more positive aspects of sexuality [67,68]. Teachers continue to perceive their responsibility as combating sexual risk, whilst viewing young people as immature and oversexualized [69], even as adolescents themselves express a preference for sex education with less emphasis on strictly negative sexual outcomes [16], and more emphasis on peer education [70].

As for more positive views of sexuality, only on rare occasions do interventions address issues such as sexual pleasure, desire and healthy relationships. Desire and pleasure were not included in the outcome evaluations for school settings, nor for digital and blended learning programs included in this review: again this is in line with the position of other authors cited in the present study, who advocate the need to also embrace the more positive aspects of sexuality [53,56]. Specifically, Bailey and colleagues [56] (p. 73) suggest as “optimal outcomes” social and emotional well-being in sexual health. Young people want to know about more than STIs, they also “want information about sexual pleasure, how to communicate with partners about what they want sexually and specific techniques to better pleasure their partners” [59] (p. 282). Similarly, Kedzior et al. [51] also argue for the need to move beyond a risk-aversion approach and towards one that places more emphasis on positive adolescent sexual and reproductive health.

Pleasure and desire are largely absent within sex and relationship education [71] and, when they are included, they are often proposed as part of a discourse on safe practice, where pleasure continues to be equated with danger [72]. The persistent absence of a “discourse of desire” in sex education [73,74] is especially problematic for women, for whom desire is still mediated by (positive) male attention, and for whom pleasure is derived from being found desirable and not from sexual self-expression or from their own desires [75]. Receiving sexualized attention from men makes women “feel good” by increasing their self-esteem and self-confidence [76]. However, it is still men who decide what is sexy and what is not, based on the attention they pay to women “girl watching”, [77] (p. 386), which leads the latter to self-objectify [78] with all the attendant negative consequences for their overall and sexual health [79]. In fact, women experience “pushes” and “pulls” [80] (p.393) with regard to sexualized culture. In one sense, the sexualization of culture has placed women in the position of subjects who desire, not just that of subjects who are desired, but at the same time it becomes a form of regulation in which young women are forced to assume the current sexualized ideal [81,82] in order to position themselves as “modern, liberated and feminine,” and avoid being seen as “outdated or prudish” [83] (p. 16). Koepsel [84] provides a holistic definition of pleasure as well as clear recommendations for how educators can overcome these deficits by incorporating pleasure into their existing curricula. At present, sexual education is still largely centered on questions of public health, and there is as yet no consensus on criteria for defining sexual well-being and other aspects of positive sexuality [85]. Patterson et al. [86] argue for the need to mandate “comprehensive, positive, inclusive and skills-based learning” to enhance people´s ability to develop healthy positive relationships throughout their lives.

The absence of desire and pleasure in the outcomes of the evaluated reviews is connected with the absence of gender-related outcomes. Only one of the reviews addresses the issue of gender and power in sexuality programs [54], illustrating how their inclusion can bring about a five-fold increase in the effectiveness of risk behavior prevention. Nonetheless, men are far less likely than women to sign up for a sexuality course, and as a result of masculine ideologies many young males experience negative attitudes towards sex education [87]. To date we still have little idea as to what are the “active ingredients” that can contribute to successfully encouraging men to challenge gender inequalities, male privilege and harmful or restrictive masculinities so as to help improve sexual and reproductive health for all [88] (p.16). Schmidt et al.’s [89] review looks at 10 evidence-based sexual education programs in schools: the majority discuss sexually transmitted diseases and unplanned pregnancy, abstinence, and contraceptive use, while very few address components related to healthy dating relationships, discussion of interpersonal violence or an understanding of gender roles.

The International Guidance on Sexuality Education [90], and the International Technical Guidance on Sexuality Education [17] promote the delivery of sexual education within a framework of human rights and gender equality to support children and adolescents in questioning social and cultural norms. The year 2020 marked the anniversaries of several path breaking policies, laws and events for women’s rights: the 100th anniversary of women´s suffrage in the United States; the 25th anniversary of the Beijing Platform for Action, a global roadmap for women´s empowerment; and, the 20th anniversary of the United Nations Security Council Resolution for a Women, Peace and Security agenda. Although there have been important advances in recent years in research relating to the inclusion of gender equality and human rights interventions in ASRH policies and programming still “fundamental gaps remain” [40] (p.14). Gender equality, and to an even greater extent human rights, have had very little presence in sexual and reproductive health programs and policies, and there is a pressing need to do more to address these issues systematically. Specifically, issues such as abortion and female genital mutilation, with clear repercussions in terms of gender equality and human rights, are rarely dealt with [40].

Furthermore, sexual education that privileges heterosexuality reinforces hegemonic attributes of femininity and masculinity, and ignores identities that distance themselves from these patterns. Our collective heteronormative legacy marginalizes and harms LGB families [91] and LGBTQ+-related information about healthy relationships is largely absent from sexual and reproductive health programs [92]. Students want a more LGBTQ+ inclusive curriculum [92]: in the present RoR one review [53] addresses the issue of non-heteronormative identity in sexuality programs with significant results; and other authors are exploring promising initiatives which are also challenging this lack of inclusivity [93] and rectifying heterosexual bias [94]. However, unfortunately, the underlying neoliberal focus of the majority of contemporary sexuality education militates to assimilate LGBTQ+ people into existing economic and social normative frameworks rather than helping disrupt them [95].

### 4.2. Effectiveness

This present review of reviews shows a variety of types of sexual health promotion initiatives across the three settings (school-based, digital and blended learning), with inconsistent results. The reviews with lower risk of bias are those carried out in school settings and those that are most critical regarding the effectiveness of programs promoting ASRH, both in the prevention of pregnancies and of HIV/STIs. Reviews dealing with digital platforms and blended learning show greater effectiveness in terms of promoting adolescent sexual health: however, these are also the studies that incorporate the highest risks of bias. Specifically, in digital platforms programs the great variety of alternatives makes comparability difficult. Moreover, these programs, along with blended learning, are in a more incipient state of evaluation, compared to school-setting evaluations, and present greater risks of lower quality than reviews in school settings.

The results of the present RoR are in line with those of previous RoRs [19,32]. The review of reviews by Denford et al.s´ [19] RoR covered 37 reviews up to 2016 and summarized 224 primary randomized controlled trials: whilst it concludes that school-based programs addressing risky sexual behavior can be effective, its reviews of exclusively school-based studies offer mixed results as to effectiveness in relation to attitudes, skills and behavioral change. Some of those studies report positive effects while others find there are no effects, if not even negative effects, in terms of the aforementioned outcomes [19]. As regards pregnancy, programs appear to be effective at increasing awareness regarding STIs and contraception but overall the findings suggest that the impact of these interventions on attitudes, behaviors and skills variables are mixed, with some studies leading to improvements whilst others show no change. Moreover, the fact that community-based programs were also taken into consideration might have led to the effectiveness of school-based programs being exaggerated [19].

However, although in our RoR the higher quality/lower bias studies—in keeping with the findings of previous reviews [19,33]—fail to show a clear pattern of effectiveness, the interventions could nevertheless be generating changes as Denford et al. [19] suggest, though not in the measured outcomes, bearing in mind the low incidence of sexual intercourse and pregnancy in school-going adolescents.

With regard to school settings, Peterson et al. [55] conclude that further, more rigorous evidence is necessary to evaluate the extent to which interventions addressing school-related factors are effective and to help better understand the mechanisms by which they may contribute to improving adolescent sexual health. With regard to digital platform programs, Wadham et al. [62] (p. 101) argue that “although new media has the capacity to expand efficiencies and coverage, the technology itself does not guarantee success.” An interesting observation in their review was that interventions which were either web-based adaptations of prior prevention programs, or were theory-based or had been developed from models of behavioral change appeared effective independently of the chosen digital media mode. However, digital programs are still in the early stages of design and evaluation, especially in terms of the effects of peer interaction and often diverge from existing theoretical models [61] (p. 13). The expert opinion-based proposal of the European Society for Sexual Medicine [96] argues that e-sexual health education can contribute to improving the sexual health of the population it seems the future of CSHE is moving towards smartphone apps [97]. 

However, “despite clear and compelling evidence for the benefits of high-quality curriculum-based CSE, few children and young people receive preparation for their lives that empowers them to take control and make informed decisions about their sexuality and relationships freely and responsibly” [17] (p. 12), and during “the current public health crisis, the sexual and reproductive health of adolescents and young adults must not be overlooked, as it is integral to both their and the larger society’s well-being” [28] (p. 9). In the light of these challenges, Coyle et al.’s [64] suggestion that the blended learning model may end up achieving a far more dominant role in the future of sexual education acquires even more relevance.

### 4.3. Limitations

This study represents the first review of reviews, as far as we are aware, in which the effectiveness of sex education programs in different settings (school-based, digital and blended learning) is evaluated, using a rich methodology and providing interesting conclusions. However, the present review of reviews is not without its limitations.

While systematic reviews and reviews of reviews can offer a way synthesizing large amounts of data, the great heterogeneity and diversity of measured outcomes make it difficult to establish a synthesis of the results, even more so in cases where it is not possible to apply meta-analysis. Furthermore, the quality of reviews of reviews is limited by that of the reviews they include and RoRs do not necessarily represent the leading edge research in the field.

In addition, although we searched for a wide range of keywords on the most commonly used databases in the field of health (namely ERIC, Web of Science, PubMed, and PsycINFO) to identify relevant papers, it is possible that the choice of keywords and database may have resulted in our omitting some relevant studies. Moreover, our review has focused on articles in international journals published in English, allowing us access to the most rigorous peer-reviewed studies and to those with greater international diffusion, given that English is the most frequently used language in the scientific environment: notwithstanding, this has also limited the scope of our review by precluding research published in other languages and contexts. Nor have documents that could have been found in the gray literature been included, given that only peer-reviewed studies have been considered for inclusion.

It is worth remembering moreover that most of the data on the outcomes of the studies included are self-reported, with mention of only occasional biological outcomes, which may limit the reliability of the effectiveness results. This represents another interesting reflection on the way in which the evaluation of the effectiveness of programs on sexual education is being carried out, and alerts us to the need for change.

Finally, it should be noted that this review of reviews is focused on adolescents from high-income countries, and our results show that studies carried out in the United States are largely overrepresented, since it is the country that provides the highest number of samples, especially in school settings: this may give rise to bias when it comes to generalizing from these results. Once again, this raises another necessary reflection on the capitalization that studies focused on American samples are having in the construction of the body of scientific knowledge on sexual and reproductive behavior, when in reality sexuality is conditioned by socio-economic variables that require a far-more multicultural and world-centric approach.

## 5. Conclusions

This review of reviews is the first to assess jointly the effectiveness of school-based, digital and blended learning interventions in ASRH in high-income countries. The effectiveness of the sex education programs reviewed mostly focused on the reduction of risky behaviors (e.g., STI or unwanted pregnancies) as public health outcomes; however, pleasure, desire and healthy relationships are outcomes that are mostly conspicuous by their absence in the reviews we have covered. Nonetheless, the broad range of studies included in this RoR, with their diversity of settings and methods, populations and objectives, precludes any easily drawn comparisons or conclusions. The inconsistent results and the high risk of bias reduce the conclusiveness of this review, so a more rigorous assessment of the effectivity of sexual education programs is pending and action needs to be taken to guarantee better and more rigorous evaluations, with sufficient human and financial resources. Schools and organizations need technical assistance to build the capacity for rigorous program planning, implementation and evaluation [98]. To this end, there are already examples of interesting proposals, such as that of the Working to Institutionalize Sex Education (WISE) Initiative, a privately funded effort to help public school districts develop and deliver comprehensive sexuality programs in the USA [99].

The extent of the risks of bias identified in the reviews and studies covered by this RoR points to an important conclusion, allowing us to highlight the precariousness that characterizes the evaluation of sexual education programs and the consequent undermining of public policy oriented to promoting ASRH. Public policies that promote ASRH are of vital importance when it comes to minimizing risks related to sexual behavior, and maximizing healthy relations and sexual well-being for the youngest members of our society.

Above all it is important to recognize the opportunities afforded by new technologies, so ubiquitous in the lives of young people, since they allow for programs that are far more cost-effective than traditional, in-person interventions. Finally, blended learning programs are perhaps even more promising, given their combination of the best of face-to-face and digital interventions, meaning they provide an excellent educative tool in the new context of the COVID-19 pandemic, and may even become the dominant teaching model in the future.

## Data Availability

The data presented in this study are available from the corresponding author on reasonable request.

## Appendix A

**Table A1:** Search Terms Used.

Characteristic	Search Terms
Sex education	“sex education” OR “sexuality education” OR “sex education program” OR “sexuality education program” OR “reproductive education” OR “Sexual health education” OR “reproductive health education” OR “sexual and reproductive health” OR “sexual health”
Study population (adolescents)	“adolescent” OR “adolescents” OR “teenagers” OR “young people” OR “young person” OR “primary students” OR “Secondary Students” OR “student”
Setting (school, online, blended learning)	“internet” OR “online” OR “offline” OR “virtual” OR “digital” OR “computer” OR “computer-technology” OR “technology” OR “computerized” OR “internet-based intervention” OR “computer based approach” OR “computer-assisted education” OR “school” OR “school-based” OR “K-12 setting” OR “school based programs” or “school setting” OR “blended learning”
Evaluation (review of reviews)	“evaluation” OR “assessment” OR “impact” OR “intervention” OR “impact evaluation” OR “outcome evaluation” OR “process evaluation” OR “comparative effectiveness research” OR “review” OR “review of reviews” OR “systematic reviews” OR “narrative reviews”

**Table A2:** Description of studies.

**School**								
**Authors/Year**	**Title**	**Country of the Review**	**Search Tools**	**Cover Period**	**Year of last Paper Included**	**Last Paper Included**	**Country of the Studies Included**	**Synthesis**
Chokprajakchad et al. (2018)	Sexual Health Interventions Among Early Adolescents: An Integrative Review.	Thailand	PubMed, CINAHL, Scopus, Science Direct, Web of Science, Thaijo and TCI.	2006–2017	2016	33 studies	International.	Narrative
Goldfarb et al. (2020)	Three Decades of Research: The Case for Comprehensive Sex Education.	USA	ERIC, Psycinfo and MEDLINE.	1990–2017	2017	80 studies	USA (*n* = 55), Israel (*n* = 1), Canada (*n* = 6), Australia (*n* = 3), New Zealand (*n* = 1), The Netherlands (*n* = 2) Kenya (*n* = 1), Mexico (*n* = 2), South Africa (*n* = 1), Ireland (*n* = 2), South Korea (*n* = 1), China (*n* = 1), Holland (*n* = 1) U.K (*n* = 1), Europe (*n* = 2).	Narrative
Haberland et al. (2016)	The Case for Addressing Gender and Power in Sexuality and HIV Education: A Comprehensive Review of Evaluation Studies.	USA	PubMed, ERIC, Cochrane Central Register of Controlled Trials and Eldis.	1990–2012	2011	22 studies	USA (*n* = 14). High income countries other than the United States (*n* = 2). Low or middle income country (*n* = 6).	Meta-analysis (one outcome) and Narrative
Kedzior et al. (2020)	A Systematic Review of School-Based Programs to Improve Adolescent Sexual and Reproductive Health: Considering The Role of Social Connectedness.	Australia	PubMed, CINAHL, Embase, Psycinfo, ERIC and SCOPUS.	July 2019	2017	18 studies	International.	Narrative
Lopez et al. (2016)	School-Based Interventions for Improving Contraceptive Use in Adolescents.	USA	PubMed, CENTRAL, ERIC, Web of Science and POPLINE.	1981–2016	2014	11 studies	USA (*n* = 6). U.K (*n* = 1). Mexico (*n* = 3). South Africa (*n* = 1).	Narrative
Marseille et al. (2018)	Effectiveness of School-Based Teen Pregnancy Prevention Programs in The USA: A Systematic Review and Meta-Analysis.	USA	Cochrane Central, ERIC, PubMed, Psycinfo, Scopus, Web of Science and The Gray Literature.	1985–2017	2016	21 studies	USA (*n* = 14). Canada (*n* = 4).	Meta-analysis
Mason-Jones et al. (2016)	School-Based Interventions for Preventing HIV, Sexually Transmitted Infections, and Pregnancy in Adolescents.	United Kingdom	MEDLINE, CENTRAL, OMS, AIDS, AEGIS, CDC, and ONUSIDA.	1990–2016	2015	8 studies	Sub-Saharan Africa: (South Africa, Tanzania Zimbabwe, Malawi Kenya) *n* = 5, Europe: (England and Scotland) *n* = 2, Latin America (*n* = 1).	Meta-analysis
Mirzazadeh et al. (2018)	Do School-Based Programs Prevent HIV and Other Sexually Transmitted Infections in Adolescents? A Systematic Review and Meta-Analysis.	USA	PubMed, Cochrane Central Register of Controlled Trials, ERIC, Psycinfo, Scopus, Web ofScience andThe Gray Literature.	May 2017	2017	9 studies	USA (*n* = 9).	Meta-analysis
Oringanje et al. (2016)	Interventions for Preventing Unintended Pregnancies Among Adolescents	USA	CENTRAL, The Cochrane Library, MEDLINE, EMBASE, LILACS, Social Science Citation Index and Science Citation Index, Dissertations Abstracts Online, Network, HealthStar, Psycinfo, CINAHL, POPLINE and The Gray Literature	1994–2015	2015	53 studies	USA (*n* = 41), England (*n* = 2), Scotland (*n* = 2), Canada (*n* = 1), Italy (*n* = 1), Mexico (*n* = 2), Low and middle income countries (*n* = 4).	Narrative
Peterson et al. (2019)	Effects of Interventions Addressing School Environments or Educational Assets on Adolescent Sexual Health: Systematic Review and Meta-Analysis.	USA	BiblioMap, CINAHL Plus, ERIC, IBSS, Open Grey, ProQuest, Psycinfo, Medline and Web of Science.	1999–2016	2016	11 studies	Australia and USA (*n* = 5), South Africa and Kenya (*n* = 4), Malawi and Zimbabwe (n = 2).	Meta-analysis and narrative
**Online**								
**Authors/Year**	**Title**	**Country of the review**	**Search tools**	**Cover period**	**Year of last paper included**	**Last paper included**	**Country of the included studies**	**Synthesis**
Bailey et al. (2015)	Sexual Health Promotion for Young People Delivered Via Digital Media: A Scoping Review.	United Kingdom	CENTRAL, DARE, MEDLINE, EMBASE, CINAHL, BNI, Psycinfo and The Gray Literature.	1989–2013	2013	19 studies	United Kingdom (*n* = 19).	Meta-analysis andNarrative
Celik et al. (2020)	The Effect of Technology-Based Programmes On Changing Health Behaviours of Adolescents: Systematic Review.	Turkey	PubMeb and Science direct databases.	2011–2016	2016	16 studies	Canada (*n* = 2), New Zealand (*n* = 1), Australia (*n* = 3), Norway (*n* = 1), USA (*n* = 9).	Narrative
Desmet et al. (2015)	A Systematic Review and Meta-Analysis of Interventions for Sexual Health Promotion Involving Serious Digital Games.	Belgium	PubMed, Web of Science, CINAHL and Psycinfo.	July 2013	2012	7 studies	USA (*n* = 6), United Kingdom (*n* = 1).	Meta-analysis
Holstrom (2015)	Sexuality Education Goes Viral: What We Know About Online Sexual Health Information.	USA	Medline, EBSCO, ERIC and PubMed. The EBSCO.	2004–2014	2012	5 studies	USA (*n* = 3), Australia (*n* = 1), Europe (*n* = 1).	Narrative
L’Engle et al. (2016)	Mobile Phone Interventions for Adolescent Sexual and Reproductive Health: A Systematic Review.	USA	PubMed, Embase, Global Health, Psycinfo, Popline, Cochrane Library, Web of Science and The Gray Literature.	2000–2014	2014	35 studies	USA (*n* = 35).	Narrative
Martin et al. (2020)	Participatory Interventions for Sexual Health Promotion for Adolescents and Young Adults on The Internet: Systematic Review.	France	PubMeb, Aurore database and The Gray Literature.	2006–2019	2019	60 studies	USA (*n* = 38), Canada (*n* = 1), United Kingdom (*n* = 4), Netherlands (*n* = 1), Europe (*n* = 2). Australia (*n* = 3), Uganda (*n* = 4), Brazil (*n* = 2), Chile (*n* = 2), Asia (*n* = 3),	Narrative
Palmer et al. (2020)	Targeted Client Communication Via Mobile Devices for Improving Sexual and Reproductive Health.	United Kingdom	Cochrane Central Register of Controlled Trials, MEDLINE, POPLINE, WHO Global Health Library and The Gray Literature.	July 2019	2017	33 studies	Colombia (*n* = 1), China (*n* = 2), Australia (*n* = 2), USA (*n* = 9), U.K. (*n* = 2), Peru (*n* = 1), Lower middle income (*n* = 16).	Meta-analysis AndNarrative
Wadham et al. (2019)	New Digital Media Interventions for Sexual Health Promotion Among Young People: A Systematic Review.	Australia	CINAHL, Medline, Psycinfo, Socindex, Informit, PubMed and Scopus.	2010–2017	2016	25 studies	USA (*n* = 16), Canada (*n* = 1), Netherlands (*n* = 2), Australia (*n* = 2), African American communities (*n* = 1), Chile (*n* = 1), Uganda (*n* = 1), Thailand (*n* = 1).	Narrative
Widman et al. (2018)	Technology-Based Interventions to Reduce Sexually Transmitted Infections and Unintended Pregnancy Among Youth.	USA	Medline, Psycinfo and Communication Source.	May 2017	2015	16 studies	USA (*n* = 16).	Meta-analysis
**Blended Learning**								
**Authors/Year**	**Title**	**Country of the Review**	**Search Tools**	**Cover Period**	**Year of last Paper Included**	**Last Paper Included**	**Country of the Included Studies**	**Synthesis**
Coyle et al. (2019)	Blended Learning for Sexual Health Education: Evidence Base, Promising Practices, and Potential Challenges.	USA	Google Scholar, PubMed and the Cumulative Index of Nursing.	2000–2017	2015	9 studies	USA (*n* = 6), U.K (*n* = 2), Europe (*n* = 1).	Narrative

**Table A3:** Characteristics and main results of the studies included.

**School**					
**Authors/Year**	**Objective**	**Participants**	**Type of Study**	**Outcomes**	**Results**
Chokprajakchad et al. (2018)	To describe and analyze methodological and substantive features of research on interventions to delay the initiation of sexual intercourse and prevent other sexual risk behaviors among early adolescents.	10–13 years	14 studies used randomized controlled trials (RCTs), 16 used quasi-experimental designs and three used a pre-test, post-test design.	*PRIMARY* (a) Adolescent sexual behavior. (b) Initiation of sexual activity. (c) Condom use and other. Contraceptive use. *SECONDARY* (a) Adolescents’ attitudes. (b) Self-efficacy. (c) Intentions related to sexual behavior.	A total of 14 studies measured only adolescent psychosocial outcomes related to sexual behavior. A total of 17 studies measured the outcomes of sexual initiation, while 18 studies measured other sexual risk behaviors such as recent sexual activity (six studies), a number of sexual partners (three studies) and contraception and/or condom use (nine studies). In total, 22 programs reviewed were effective in changing targeted adolescent psychosocial and/or behavioral outcomes. Many of the studies reviewed demonstrated impacts on short-term outcomes, such as knowledge, attitudes, perception and intention. Delay in the initiation of sexual intercourse, the sexual behavior most commonly measured by studies in this review, was seen in 12 of 17 studies evaluating this outcome.
Goldfarb et al. (2020)	To find evidence for the effectiveness of comprehensive sex education in school-based programs.	3–18 years	Randomized controlled trial (RCTs), quasi-experimental, and pre- and post-test.	*PRIMARY* *(a) Appreciation of sexual diversity:* Homophobia, homophobic bullying, understanding of gender/gender norms, recognition of gender equity, rights, and social justice. *(b) Dating and intimate partner violence prevention:* Knowledge and attitudes about, and reporting of, DV and IPV; DV and IPV perpetration and victimization; bystander, intentions and behaviors. *(c) Healthy Relationships.* Knowledge, attitudes, and skills and intentions. *(d) Child sex abuse prevention:* Knowledge, attitudes, skills and social-emotional outcomes related to personal safety and touch. *(e) Additional outcomes* Social emotional learning. Media literacy.	Appreciation of Sexual Diversity: lower homophobia, reduced homophobic bullying, expanded understanding of gender/gender norms, recognition of gender equity, rights, and social justice. Dating and Intimate Partner Violence prevention: improved knowledge and attitudes about, and reporting of, DV and IPV, decreased DV and IPV perpetration and victimization, increased bystander intentions and behaviors. Healthy relationships: increased knowledge, attitudes and skills, improved communication skills and intentions. Child Sex Abuse prevention: improved knowledge, attitudes, skills and social-emotional outcomes related to personal safety and touch, improved disclosure and behaviors). Additional outcomes: social-emotional learning and media literacy.
Haberland et al. (2016)	Evaluation of behavior-change interventions to prevent HIV, STIs or unintended pregnancy to analyze whether addressing gender and power in sexuality education curricula is associated with better outcomes.	Adolescents under 19 years	Randomized Controlled Trials (RCTs) or quasi-experimental.	*PRIMARY* (a) STIs. (b) HIV. (c) Pregnancy. (d) Childbearing.	Of the 22 interventions that met the inclusion criteria, 10 addressed gender or power, and 12 did not. The programs that addressed gender or power were five times as likely to be effective (positive effects on sexual and reproductive health—including knowledge, attitudes, reported behavior change and health outcomes) as those that did not; in all 80% of them were associated with a significantly lower rate of STIs or unintended pregnancy. In contrast, among the programs that did not address gender or power, only 17% had such an association.
Kedzior et al. (2020)	Determine the impact of school-based programs that promote social connectedness on adolescent sexual and reproductive health.	10–19 years	Randomized controlled trials, non-randomized controlled trials (including quasi), controlled before-after (pre-/post-) interrupted time series, and program evaluations. Program evaluation without a control group were eligible if they reported on outcomes pre- and post- program implementation.	*PRIMARY* (a) Contraception use. (b) Intercourse (frequency or another outcome as defined by authors). (c) Risk of adolescent pregnancy and birth. (d) Rates of sexually transmissible infections (STIs). (e) Attitudes, beliefs and knowledge about sex and reproductive health. (f) Autonomy. (g) Connectedness.	Improved condom use, delayed initiation of sex, and reduced pregnancy rates. Program effectiveness was influenced by ethnicity and gender: greater improvements in condom use were often reported among African American students. Programs that were most effective incorporated multiple constructs of social connectedness, included social skill-building and had a sustained intensity.
Lopez et al. (2016)	To identify school-based interventions that improved contraceptive use among adolescents.	19 years or younger	Randomized controlled trials (RCTs). (Of 11 trials, 10 were cluster randomized).	*PRIMARY* (a) Pregnancy (six months or more after the intervention began). (b) Contraceptive use (three months or more after the intervention began). *SECONDARY* (a) Knowledge of contraceptive effectiveness or effective method use. (b) Attitude about contraception or a specific contraceptive method.	Of the trials included, most compared the new programs to ‘usual’ sex education. Many trials assessed contraceptive use as an outcome but did not report whether the content included contraceptive methods and their relative effectiveness. Since most trials aimed to prevent STI/HIV and pregnancy, they focused on condom use. However, several studies covered a variety of birth control methods. The overall quality of results was low: some trials lacked information on how their programs worked, many analyzed subsamples rather than all students in the study, and most had high losses.
Marseille et al. (2018)	To evaluate the effectiveness of school-based teen pregnancy prevention programs in the USA.	10–19 years	Randomized controlled trials (RCTs) (10 studies) and non-RCTs (11 studies) with comparator groups were eligible yielded 30 unique pooled comparisons for pregnancy.	*PRIMARY* Pregnancy. *SECONDARY* (a) Sexual Initiation. (b) Condom Use. (c) Oral Contraception Pill Use.	Regarding primary outcomes: 30 unique pooled comparisons for pregnancy were included, of which 24 were not statistically significant and 6 showed statistically significant changes in pregnancy rates (two with increased risk and four with decreased risk) Regarding the secondary outcomes: the majority of the pooled risk reduction ratios were not statistically significant. No consistent evidence of increasing condom or OCP use, or delaying sexual initiation were found. The six that were statistically significant for sexual initiation showed a reduced risk of sexual initiation as did the four for no condom use. All studies were at high risk of bias and the quality of evidence was low or very low.
Mason-Jones et al. (2016)	To evaluate the effects of school-based sexual and reproductive health programs on sexually transmitted infections (such as HIV, herpes simplex virus, and syphilis), and pregnancy among adolescents.	10–19 years	Randomized Controlled Trials (RCTs) (both individually randomized and cluster-randomized included 8 cluster-RCTs).	*PRIMARY* *Clinical/biological outcomes**:* (a) HIV prevalence. (b) STI prevalence. (c) Pregnancy prevalence. *Behavioral self-reported outcomes:* (a) Use of male condoms at first sex. (b) Use of male condoms at most recent (last) sex. (c) Initiation (sexual debut).	The educational programs evaluated had no demonstrable effect on the prevalence of HIV (low certainty evidence), or other sexually transmitted infections (Herpes Simplex virus prevalence: moderate certainty evidence; Syphilis prevalence: low certainty evidence). There was also no apparent effect on the number of young women who were pregnant at the end of the trial (moderate certainty evidence). Combined educational and incentive-based programs herpes simplex virus infection was reduced, predominantly in young women, but no effect was detected for HIV or pregnancy (low certainty evidence). It was not possible to show effectiveness for educational curriculum-based interventions on biologically measured adolescent sexual and reproductive health outcomes.
Mirzazadeh et al. (2018)	To evaluate the effectiveness of school-based programs prevent HIV and other sexually Transmitted Infections in adolescents in the USA.	10–19 years	Three RCTs and six non-RCTs describing seven interventions.	*PRIMARY* (a) HIV/STI incidence or prevalence. (b) HIV/STI testing. *SECONDARY* (a) Frequency of intercourse. (b) Number of partners. (c) Initiation of sexual intercourse. (d) Sex without a condom. (e) HIV/STI knowledge, attitude, and behavior.	Of the eight studies reviewed, only two studies published from one intervention that had an effect on the primary outcome of interest, STI incidence, and none that reported HIV incidence. No studies that assessed the effect of school-based prevention programs on HIV incidence among adolescents were found. The only effective intervention seemed to be one that covered multiple years, started early, and had multiple components. The quality of evidence for all outcomes was very low. Studies, including the RCTs, were of low methodological quality and had mixed findings, thus offering no persuasive evidence for the effectiveness of school-based programs. While some positive effects on changes in STI-related knowledge and attitudes were found, there was little evidence that these changes decrease STI. The variability in the interventions, study populations, settings, and outcomes reviewed make it difficult to identify the specific aspects of an intervention that may be most effective at reducing STIs and HIV among young people.
Oringanje et al. (2016)	To assess the effects of primary prevention interventions (school-based, community/home-based, clinic-based, and faith-based) on unintended pregnancies among adolescents.	10–19 years	53 Randomized Controlled Trials (RCTs) comparing these interventions to various control groups (mostly usual standard sex education offered by schools).	*PRIMARY* (a) Unintended pregnancy. *SECONDARY* (a) Reported changes in knowledge and attitudes about the risk of unintended pregnancies. (b) Initiation of sexual intercourse. (c) Use of birth control methods. (d) Abortion. (e) Childbirth. (f) Morbidity related to pregnancy, abortion or child birth. (g) Mortality related to pregnancy, abortion or childbirth. (h) Sexually transmitted infections (including HIV).	Only interventions involving a combination of education and contraception promotion (multiple interventions) were seen to significantly reduce unintended pregnancy over the medium-term and long-term follow-up period. Evidence for program effects on biological measures is limited. Results for behavioral (secondary) outcomes were inconsistent across trials. The variability in study populations, interventions and outcomes of included trials, and the paucity of studies directly comparing different interventions preclude a definitive conclusion regarding which type of intervention is most effective. Limitations include reliance on program participants to report their behaviors accurately and methodological weaknesses in the trials.
Peterson et al. (2019)	To examine whether interventions, addressing school-level environment or student-level educational assets, can promote young people’s sexual health.	10–19 years	Randomized trial or quasi experimental design, in which control groups received usual treatment or a comparison intervention, and they must have reported at least one sexual health outcome, such as pregnancy, STDs or sexual behaviors associated with increased risk of pregnancy or STDs.	*PRIMARY Interventions designed specifically to improve:* (a) Knowledge. (b) Attitudes. (c) Skills. (d) Services related to sexual health.	The meta-analysis of three randomized trials provided some evidence that school-environment interventions may delay sexual debut (pooled odds ratio, 0.5). Narrative synthesis of the remaining outcomes found mixed results, but suggests that interventions addressing school-level environment may delay sexual debut and that those addressing student-level educational assets may reduce risk of pregnancy and STDs.
**Online**					
**Authors/** **Year**	**Objective**	**Participants**	**Type of Study**	**Outcomes**	**Results**
Bailey et al. (2015)	To summarize evidence on effectiveness, cost-effectiveness and mechanism of action of interactive digital interventions (IDIs) for sexual health; optimal practice for intervention development; contexts for successful implementation; research methods for digital intervention evaluation; and the future potential of sexual health promotion via digital media.	12–19 years	Randomized controlled trials (RCTs).	*PRIMARY* (a) Sexual health knowledge. (b) Self-efficacy. (c) Intention/motivation. (d) Sexual behavior and biological.	Interactive digital interventions are effective tools for learning about sexual health. Interactive digital interventions have small but significant effects on self-efficacy, and sexual behavior. There is not enough evidence to be sure of the effects on biological outcomes or to be sure of longer-term impacts. *Effectiveness of interactive digital interventions effective compared with minimal interventions.* Significant, moderate effect on sexual health knowledge. A small but significant effect on self-efficacy. No demonstrable effect on sexual behavior and on STI diagnoses. *Effectiveness of interactive digital interventions compared to face-to-face sexual health interventions.* Significant, moderate positive effect on sexual health knowledge. No demonstrable effect on self-efficacy. Small, significant effect on intention.
Celik et al. (2020)	To determine the effect of technology-based programmes in changing adolescent health behaviors.	10–24 years	Randomized control group.	*PRIMARY* Adolescents’ health-promoting behaviors: pregnancy, HIV/disease-related knowledge, condom use, condom intentions, condom skills, self-efficacy, and related infectious diseases risk behavior.	A statistically significant increase was determined in health-promoting behavior in one (Marsch et al., 2011) of four studies on sexual health. In 56.25% of the studies, the development in the studied health behaviors was found to be significant.
Desmet et al. (2015)	To analyze the effectiveness of interventions for sexual health promotion that use serious digital games.	13–29 years	Randomized control group, and randomized on an individual.	*PRIMARY* Behavior, knowledge, behavioral intention, perceived environmental constraints, skills, attitudes, subjective norm, and self-efficacy. *SECONDARY* Clinical effects (e.g., rates of sexually transmitted infections).	Interventions for sexual health promotion using serious games have significant positive effects for determinants, albeit rather small. The effects on behavior, measured in only two studies, were not significant. Most games did not use many immersive game features. Instead, there was a strong reliance on pure gamification features such as reward and feedback.
Holstrom (2015)	To draw a more comprehensive picture of how online sexual health interventions do and do not align with real world habits and interests of adolescents.	10–24 years	Randomized controlled trials (RCTs), and focus groups participants.	*PRIMARY* (a) Sexual Health information. (b) What topics they want to know about. (c) Evaluations of Internet-based sexual health interventions.	Intervention exposure was associated with increased sexual health knowledge and awareness, lower rates of unprotected sex and higher rates of condom use, and greater STI testing. First, it is worth trying to replicate and continue evaluating the interventions that yielded modest results. Second, online sexual health education is lacking consensus on what is a successful outcome, how to measure it, or what theoretical foundations should be used to build interventions. Third, the evaluated interventions do not echo some primary components of what we know adolescents want from a sexual health website.
L’Engle et al. (2016)	To assess strategies, findings, and quality of evidence on using mobile phones to improve adolescent sexual and reproductive health (ASRH).	13–24 years	Randomized controlled trials (RCTs), quasi-experimental, observational, or descriptive research.	*PRIMARY* (a) Promote positive and preventive SRH behaviors. (b) Increase adoption and continuation of contraception. (c) Support medication adherence for HIV-positive young people. (d) Encourage use of health screening and treatment services.	Evidence on mobile phone interventions for ASRH published in peer-reviewed journals reflects a high degree of quality in methods and reporting. Improved SRH knowledge, less unprotected sex and more STI testing. Leveraging mobile phones to increase youth contact for STI screening and follow-up yielded higher rates of screening and recall and more timely and complete STI treatment and vaccination. Increased adolescent patient adherence to medication (oral contraceptive pills and SRT) in USA. Using mobile phone calls to provide adolescent patient counseling was ineffective, except for 1 small study. Mobile phones were used to increase health program reach to adolescents and ethnic and minority subgroups, to increase confidentiality in providing sensitive SRH information to young people, and to provide a supportive “friend in your pocket” who reminds and encourages good health.
Martin et al. (2020)	To describe existing published studies on online participatory intervention methods used to promote the sexual health of adolescents and young adults.	10–24 years	16 Randomized Controlled Trial (RCT), 15 Control group (NI = 2), 4 Information-only control website, 7 Before-after study (no RCT), 3 Cross-sectional study, 8 other design, 3 Unspecified.	*PRIMARY* *Process outcomes evaluated:* Acceptability, Attractiveness, Feasibility, Satisfaction and Implementation. *Outcomes evaluation conducted:* Behaviors. Condom use, condom use intention, self-efficacy toward condom use, and attitude toward condom use attitudes. Communication. Knowledge. Behavioral skills. Self-efficacy. Contraception use. History of sexually transmitted infections. HIV stigma. HIV test history (date and result of the last test). Incidence of sexually transmitted infections. Intentions related to risky sexual activity. Internalized homophobia. Intimate partner violence. Motivation. Pubertal development. Sexual abstinence. Waiting before having sex.	Effectiveness results (*n* = 23) Online peer interaction, the major participatory component, is not sufficiently conceptualized and defined as a determinant of change or theoretical model component. Still in the early stages of design and evaluation, particularly as regards the effect of peer interaction, and do not always adhere to existing theoretical models. Participatory online interventions for young people’s sexual health have shown their feasibility, practical interest, and attractiveness, but their effectiveness has not yet been sufficiently evaluated.
Palmer et al. (2020)	To assess the effects of targeted client communication via delivered via mobile devices on adolescents’ knowledge, and on adolescents’ and adults’ sexual and reproductive health behavior, health service use, and health and well-being.	10 -24 years	Randomized controlled trials (RCTs).	*PRIMARY* *Health behavior change:* • STI/HIV prevention. • STI/HIV treatment. • Contraception/family planning. • Pre-conception care. • Partner violence. *Service utilization:* • STI/HIV prevention/treatment. • Contraception/family planning. • HPV vaccination. • Cervical screening. • Pre-conception care. *Partner violence:* • Use of services designed for those who have experienced partner violence. *Health status and well-being:* • STI/HIV prevention. • STI/HIV treatment. • Contraception/family planning. • Partner violence. • Well-being. *Any measure of knowledge or attitudes relating to the following:* • STI prevention and/or treatment. • Contraception/family planning. • Cervical cancer screening. • Sexual violence. • HPV vaccination. • Puberty. *SECONDARY* •Patient/client acceptability and satisfaction with the intervention. •Resource use, including cost to the system and unintended consequences.	TCCMD (Targeted client communication (TCC) delivered via mobile devices (MD)) versus standard care TCC may increase sexual health knowledge (risk ratio (RR) 1.45, 95% confidence interval (CI) 1.23 to 1.71; low-certainty evidence). TCCMD may modestly increase contraception use (RR 1.19, 95% CI 1.05 to 1.35; low-certainty evidence). The effects on condom use, antiretroviral therapy (ART) adherence, and health service use are uncertain due to very low-certainty evidence. The effects on abortion and STI rates are unknown due to lack of studies. TCCMD versus non-digital TCC (e.g., pamphlets) The effects of TCCMD on behavior (contraception use, condom use, ART adherence), service use, health and wellbeing (abortion and STI rates) are unknown due to lack of studies for this comparison. TCCMD versus digital non-targeted communication The effects on sexual health knowledge, condom and contraceptive use are uncertain due to very low-certainty evidence. Interventions may increase health service use (attendance for STI/HIV testing, RR 1.61, 95% CI 1.08 to 2.40; low-certainty evidence). The intervention may be beneficial for reducing STI rates (RR 0.61, 95% CI 0.28 to 1.33; low-certainty evidence), but the confidence interval encompasses both benefit and harm. The effects on abortion rates and on ART adherence are unknown due to lack of studies. We are uncertain whether TCCMD results in unintended consequences due to lack of evidence. There was evidence of a modest beneficial intervention effect on contraceptive use among adolescent and adult populations, but there was not sufficient evidence to demonstrate that this translated into a reduction in contraception.
Wadham et al. (2019)	To assess the effectiveness of sexual health interventions delivered via new digital media to young people.	12–24 years	Randomized to a control group and pre-/post-test evaluation design, uncontrolled longitudinal studies and the remaining studies comprised a mixture of qualitative cohort, observational and mixed methods.	*PRIMARY* (a) Behavior (number of sexual partners, number of unprotected sexual acts, frequency of condom use, negotiation skills for condom use, sex under the influence of alcohol and other drugs, testing seeking behavior). (b) Self-efficacy (condom use). (c) Skills and Abilities (sexual communication and risk assessment). (d) Intentions (to use condoms). (e) Attitudes. (f) Knowledge (HIV, STI, general sexual health). (g) Efficacy of the Intervention (feasibility, acceptability, usability, satisfaction). (h) Well-being (mental health, sexuality, self-acceptance).	A large proportion of studies (11/25) specifically focused on HIV prevention. Three interventions reported non-significant effect in condom use, two interventions reported an increase in condom use and another study reported a significant increase in self-efficacy related to condom usage. Seven studies found a statistically significant effect of the intervention on knowledge levels regarding the prevention HIV and other STI, as well as general sexual health knowledge, but only one-fifth of interventions evaluating intentions to use condoms reported significant effects due to the intervention. Of the 12 studies evaluating knowledge-based outcomes, seven found a significant effect. Of the four studies that evaluated sexual communication, only one reported a significant effect. The broad range of studies included in this review, with their diversity of methods, populations and objectives, precludes any easily drawn comparisons or conclusions.
Widman et al. (2018)	To synthesize the technology-based sexual health interventions among youth people to determine their overall efficacy on two key behavioral outcomes: condom use and abstinence.	13–24 years	Randomized to a control group and experimental or quasi-experimental design.	*PRIMARY* (a) Condom use (b) Abstinence. *SECONDARY* (a) Safer sex attitudes. (b) Social norms for safer sexual activity. (c) self-efficacy. (d) Behavioral intentions to practice safer sex. (e) Sexual health knowledge.	There was a significant weighted mean effect of technology-based interventions on condom use (d = 0.23, 95% confidence interval [CI] [0.12, 0.34], *p* < 0.001) and abstinence (d = 0.21, 95% CI [0.02, 0.40], p = 0.027). Effects did not differ by age, gender, country, intervention dose, interactivity, or program tailoring. Effects were stronger when assessed with short-term (1–5 months) than with longer term (greater than 6 months) follow-ups. Compared with control programs, technology-based interventions were also more effective in increasing sexual health knowledge (d = 0.40, *p* < 0.001), safer sex norms (d = 0.15, *p* = 0.022), and attitudes (d = 0.12, *p* = 0.016)
**Blended Learning**					
**Authors/Year**	**Objective**	**Participants**	**Type of Study**	**Outcomes**	**Results**
Coyle et al. (2019)	To identify sexual health education studies using blended learning to summarize the best practices and potential challenges.	13–24 years, and adults of over 25	Randomized Controlled Trials (RCTs).	*PRIMARY* (a) Initiation of sexual intercourse (vaginal, oral or anal intercourse). (b) Other sexual risk behaviors (condom use, communication, condom use skills, frequency of sex, unprotected sex, number of partners with whom had sex without protection, frequency of using alcohol and or other substances during sex). (c) Sexual coercion or dating violence (sexual coercion, dating violence). (d) Sexuality-related psychosocial factors (attitudes, beliefs, perceptions regarding abstinence, and protection). (e) Perceived satisfaction and usability (of blended learning).	Blended learning approaches are being used successfully in sexual health education programs, including school-based programs, and have yielded positive behavioral and psychosocial changes. Blended learning approaches are viable for sexual health education and offer numerous advantages over group-based only programs, such as confidential personalization and an instructional approach that is familiar and engaging for participants.

**Table A4:** Evaluation of the studies included (AMSTAR II).

School																	
Authors	1 ^1^	2	3	4	5	6	7	8	9	10	11	12	13	14	15	16	Overall Rating ^2^
Chokprajakchad et al. (2018)	Y	N	Y	Y	N	N	N	Y	N	N	NM	NM	N	Y	NM	N	CL
Goldfarb et al. (2020)	Y	Y	N	Y	Y	Y	Partial Y	Y	N	N	NM	NM	N	Y	NM	Y	CL
Haberland et al. (2016)	Y	Y	Y	Y	N	N	N	Partial Y	N	N	NM	NM	N	Y	NM	N	CL
Kedzior et al. (2020)	Y	Y	Y	Y	Y	Y	Partial Y	Y	Y	N	NM	NM	Y	Y	NM	Y	M
Lopez et al. (2016)	Y	Y	Y	Y	Y	Y	Y	Y	Y	Y	NM	NM	Y	Y	NM	Y	H
Marseille et al. (2018)	Y	Y	Y	Y	Y	Y	Y	Y	Y	N	Y	Y	Y	Y	Y	Y	H
Mason-Jones et al. (2016)	Y	Y	Y	Y	Y	Y	Y	Y	Y	Y	Y	Y	Y	Y	Y	Y	H
Mirzazadeh et al. (2018)	Y	Y	Y	Y	Y	Y	Y	Y	Y	N	Y	Y	Y	Y	Y	Y	H
Oringanje et al. (2016)	Y	Y	Y	Y	Y	Y	Y	Y	Y	N	NM	NM	Y	Y	NM	Y	H
Peterson et al. (2019)	Y	Y	Y	Y	Y	Y	N	Y	Y	N	Y	Y	Y	Y	Y	N	L
**Online**																	
Bailey et al. (2015)	Y	Y	Y	Y	Y	Y	N	Y	Y	N	Y	Y	Y	Y	Y	Y	L
Celik et al. (2020)	Y	Y	Y	N	N	N	Y	Y	N	N	NM	NM	N	Y	NM	Y	CL
DeSmet et al. (2015)	Y	Partial Y	Y	Y	Y	Y	N	Y	Partial Y	N	Y	Y	Y	Y	N	Y	CL
Holstrom (2015)	N	N	N	Y	N	N	N	Y	N	N	NM	NM	N	N	NM	N	CL
L´Engle et al. (2016)	Y	Y	Y	Y	Y	Y	Partial Y	Partial Y	N	Y	NM	NM	N	Y	NM	Y	CL
Martin et al. (2020)	Y	Y	Y	Y	Y	Y	Y	Y	N	N	NM	NM	N	Y	NM	Y	CL
Palmer et al. (2020)	Y	Y	Y	Y	Y	Y	Y	Y	Y	Y	Y	Y	Y	Y	Y	Y	H
Wadham et al. (2019)	N	Y	Y	Y	Partial Y	Partial Y	N	Y	N	N	NM	NM	N	N	NM	Y	CL
Widman et al. (2018)	Y	Y	Y	Y	Y	Y	Partial Y	Partial Y	Y	N	Y	Y	N	Y	Y	Y	L
**Blended Learning**																	
Coyle et al. (2019)	Y	N	N	Y	N	N	N	Y	N	N	NM	NM	N	Y	NM	N	CL

^1^ 1. Did the research questions and inclusion criteria for the review include the components of PCIO?; 2. Did the report of the review contain an explicit statement that the review methods were established prior to the conduct of the review and did the report justify any significant deviations from the protocol?; 3. Did the review authors explain their selection of the study designs for inclusion in the review?; 4. Did the review authors use a comprehensive literature search strategy?; 5. Did the review authors perform study selection in duplicate?; 6. Did the review authors perform data extraction in duplicate?; 7. Did the review authors provide a list of excluded studies and justify the exclusions?; 8. Did the review authors describe the included studies in adequate detail?; 9. Did the review authors use a satisfactory technique for assessing the risk of bias (RoB) in individual studies that were included in the review?; 10. Did the review authors report on the sources of funding for the studies included in the review?; 11. If meta-analysis was performed, did the review authors use appropriate methods for statistical combination of results?; 12. If meta-analysis was performed, did the review authors assess the potential impact of RoB in individual studies on the results of the meta-analysis or other evidence synthesis?; 13. Did the review authors account for RoB in primary studies when interpreting/discussing the results of the review?; 14. Did the review authors provide a satisfactory explanation for, and discussion of, any heterogeneity observed in the results of the review?; 15. If they performed quantitative synthesis did the review authors carry out an adequate investigation of publication bias (small study bias) and discuss its likely impact on the results of the review?; 16. Did the review authors report any potential sources of conflict of interest, including any funding they received for conducting the review? ^2^ H = Hight; M = Media; C = Low; CL = Critically Low. N = No; Y = Yes.
